# Alteration of N6-Methyladenosine RNA Profiles in Cisplatin-Induced Acute Kidney Injury in Mice

**DOI:** 10.3389/fmolb.2021.654465

**Published:** 2021-07-09

**Authors:** Can-Ming Li, Ming Li, Wen-Bo Zhao, Zeng-Chun Ye, Hui Peng

**Affiliations:** Department of Nephrology, Third Affiliated Hospital of Sun Yat-sen University, Guangzhou, China

**Keywords:** acute kidney injury, N6-methyladenosine (m6A), RNA, cisplatin, microarray

## Abstract

**Aim:** To identify the alterations of N6-methyladenosine (m^6^A) RNA profiles in cisplatin-induced acute kidney injury (Cis-AKI) in mice.

**Materials and Methods:** The total level of m^6^A and the expression of methyltransferases and demethylases in the kidneys were measured. The profiles of methylated RNAs were determined by the microarray method. Bioinformatics analysis was performed to predict the functions.

**Results:** Global m^6^A levels were increased after cisplatin treatment, accompanied by the alterations of Mettl3, Mettl14, Wtap, Fto, and Alkbh5. A total of 618 mRNAs and 98 lncRNAs were significantly differentially methylated in response to cisplatin treatment. Bioinformatics analysis indicated that the methylated mRNAs predominantly acted on the metabolic process.

**Conclusion:** M^6^A epitranscriptome might be significantly altered in Cis-AKI, which is potentially implicated in the development of nephrotoxicity.

## Background

Acute kidney injury (AKI), characterized by the abrupt loss of renal function, is a common and severe disorder in hospitalized patients (Susantitaphong et al., 2013). AKI has become a major public health concern due to its high morbidity, mortality, and healthcare costs (Lameire et al., 2013; Mehta et al., 2015; Mehta et al., 2016). The etiology of AKI is usually multifactorial and complex. Among them, renal hypoperfusion, sepsis, and nephrotoxin exposure are often the three leading causes (Ronco et al., 2019). Nephrotoxic medications have been reported to be responsible for approximately one-quarter of AKI cases and remain a critical and increasing cause of AKI both in patients who are hospitalized and in the community, especially those with cancer (Bonventre et al., 2010; Loghman-Adham et al., 2012; Rosner and Perazella, 2017; Ronco et al., 2019; Tanase et al., 2019).

Cisplatin is a chemotherapy medication that is widely used in the treatment of multiple malignant tumors (Linkermann et al., 2014; Tanase et al., 2019). Due to the pharmacokinetic characteristics of cisplatin, its metabolite concentrations within the kidney are fivefold higher than in blood, suggesting an active accumulation of drugs by intrinsic renal cells, especially in kidney tubular epithelial cells (Bajorin et al., 1986; Miller et al., 2010; Xiang et al., 2019). An increased concentration of cisplatin in cells causes damage to nuclear and mitochondrial DNA, generates reactive oxygen species (ROS), which activated pathways of apoptosis and necrosis, and consequently contributes to an episode of nephrotoxicity (Miller et al., 2010). Therefore, the dose of cisplatin is greatly limited, thus leading to impaired therapeutic efficacy in patients with cancer (Dos Santos et al., 2012; Perazella, 2012; Tanase et al., 2019; Xiang et al., 2019). Although considerable progress has been made in the last few decades on the treatment of cisplatin-induced AKI (Cis-AKI), there are still no established pharmacotherapies that are available, mostly owing to the unclear molecular mechanisms of this disease (Agarwal et al., 2016; Ronco et al., 2019; Uber and Sutherland, 2019).

Recently, accumulating evidence suggest that epigenetic regulation plays a crucial role in a myriad of kidney diseases, including renal cell carcinoma, chronic kidney disease (CKD), and AKI (Guo et al., 2019; Kato and Natarajan, 2019; Linehan and Ricketts, 2019; Ruiz-Ortega et al., 2020). Epigenetic modifications, defined as stable and heritable alterations of gene expression without changing the primary DNA sequence, are essential for a variety of physiological processes, including DNA transcription, gene expression, cellular differentiation, cell cycle, and apoptosis (Quintanilha et al., 2019). Other than DNA methylation, histone modification, and noncoding RNAs, reversible modifications of RNAs have only recently emerged as an additional regulatory layer contributing to the complexity of epigenetic regulation (Vu et al., 2019). To date, at least 170 chemical modifications have been identified in various types of RNAs in all living organisms (Boccaletto et al., 2018; Huang et al., 2020). Notably, *N* ^*6*^-methyladenosine (m^6^A), first identified in the 1970s following the discovery of the polyadenosinic acid (Poly A) structure of messenger RNA (mRNA), has been considered as the most prevalent and abundant posttranscriptional modification within eukaryotic mRNAs, microRNAs (miRNAs), long noncoding RNAs (lncRNAs), and circular RNAs (circRNAs) (Desrosiers et al., 1974; Perry and Kelley, 1974; Rottman et al., 1974; Yang et al., 2017; Chen et al., 2019). m^6^A modifications, which fall within a consensus motif RRACH (R = G or A; H = A, C, or U; A = adenine and C = cytosine), are mainly enriched in the 3′-untranslated regions (UTRs) around the stop codons of mRNAs and play a critical role in mRNA processing events, such as splicing, nuclear export, degradation, and translation (Dominissini et al., 2012; Chen et al., 2019; Livneh et al., 2020). The biological effects of m^6^A modifications are collaboratively controlled by “writer,” “eraser,” and “reader” proteins (Vu et al., 2019). Methyltransferase like 3 (Mettl3) (Schumann et al., 2016), methyltransferase like 14 (Mettl14) (Liu et al., 2014), and Wilms’ tumor 1– associating protein (Wtap) (Ping et al., 2014) are essential components of the core writer complex, which efficiently catalyzes methyl group transfer to the N6 site of adenine (Wang X. et al., 2016). The m^6^A mark can then be recognized by reader proteins, such as YT521-B homology (YTH) *N* ^*6*^-methyladenosine RNA-binding proteins (YTHDF), IGF2 mRNA-binding proteins (IGF2BPs), eukaryotic translation initiation factor 3 (eIF3), and heterogeneous nuclear ribonucleoprotein (HNRNP) protein families, and erased by RNA demethylases, including fat mass and obesity-associated protein (Fto) and AlkB homolog 5 (ALKHB5) (Jia et al., 2011; Zheng et al., 2013; Chen et al., 2019). Recent evidence suggests that abnormal mRNA m^6^A modification is linked to many diseases such as obesity, infertility, and cancer (Wei et al., 2017).

In the field of kidney diseases, it has been observed that Fto, the first-identified RNA demethylase, is abundant in the kidney and regulates the fibrogenic process through the TGF-β signaling pathway in obstructive nephropathy (Jia et al., 2011; Wang C. Y. et al., 2016). Another recent study indicated that Mettl14 played a detrimental role in renal ischemia-reperfusion injury (IRI) *via* suppressing YAP1-TEAD signaling (Xu Y. et al., 2020). In addition, data from Li X et al*.* found a strong relationship between m^6^A modification and the severity of renal interstitial fibrosis induced by unilateral ureteral obstruction (Li X. et al., 2020). Furthermore, recent evidence suggested that the m^6^A site of MALAT1 was methylated by Mettl3 and, thus, promoted the TGF-β1–induced epithelial–mesenchymal transition (EMT) in HK2 cells (Liu P. et al., 2020). Therefore, it is reasonable to believe that the m^6^A modification might be involved in the process of kidney injury. However, much less is known about the functional role and regulatory mechanism of the m^6^A modification in Cis-AKI. To fulfill this end, we explore the relationship between m^6^A modification of RNAs and Cis-AKI by the microarray technique in this study.

## Methods

### Animals

129 male mice aged about 10 weeks and weighing approximately 20–25 g were purchased from Beijing Vital River Laboratory Animal Technology Co., Ltd. (China). All mice were maintained in a temperature-controlled room under a 12 h light–dark schedule and received food and water *ad libitum*. The mice in the Cis-AKI group (four mice per group) were injected intraperitoneal with a single dose of cisplatin (20 mg/kg body weight; Selleck Chemicals, TX, United States) that was dissolved in saline as described previously (Deng et al., 2019; Li et al., 2019; Li M. et al., 2020). Control mice (four mice per group) received saline only. The animals were euthanized with chloral hydrate (400 mg/kg) at 3 days after administration of cisplatin, and blood and kidney tissue with adherent fat and connective tissue removed were collected for the subsequent detections. All protocols had received prior approval from the Animal Care Committee of Sun Yat-sen University.

### Renal Function Assessment

Renal function was evaluated by performing serum creatinine and blood urea nitrogen (BUN) assays with an automatic biochemical analyzer according to the manufacturer’s instructions (Chemray-240, Rayto Life and Analytical Sciences Co., Ltd, China).

### Histopathology

Kidney tissues were fixed in 4% paraformaldehyde, embedded in paraffin wax, and cut into 4-μm–thick tissue sections. The kidney sections were deparaffinized, rehydrated, and stained with periodic acid–Schiff (PAS) by treating with periodic acid for 5 min and covering with Schiff’s reagent for 15 min at room temperature. The average percentage of cellular casts and necrosis in at least 20 random fields (×40) was examined and used as an indicator of tubular injury. The histopathological changes were scored in a single-blind manner.

### Apoptosis Analysis

Apoptotic cell death in kidney sections was determined by the terminal deoxynucleotidyl transferase–mediated dUTP nick-end labeling (TUNEL) method with an *in situ* Cell Death Detection Kit (Roche Molecular Biochemicals, Germany) according to the manufacturer’s protocol. Positive staining was detected in the nucleus by using a fluorescence microscope (Nikon, Japan). The average number of TUNEL-positive cells was calculated in 20 randomly selected regions by a blinded investigator.

### Western Blot Analysis

The frozen kidney tissue specimens were weighted, cut into small pieces, homogenized by using a Dounce glass tissue homogenizer in ice-cold radioimmunoprecipitation assay (RIPA) buffer (Thermo Fisher, MA, United States), and supplemented with protease inhibitors (Thermo Fisher, MA, United States). Total protein was extracted from the kidney homogenate by centrifugation at 14,000 g for 15 min at 4°C and quantified with the BCA Protein Assay Kit (Beyotime biotechnology, China). Protein samples were boiled in 1× NuPAGE LDS Sample Buffer (Thermo Fisher, MA, United States) and electrophoresed in 10% SDS-PAGE gels. The proteins in the gel were then transferred to polyvinylidene difluoride (PVDF) membranes (Roche, United States) *via* a wet blotting apparatus (Bio-Rad, CA, United States). These membranes were blocked in a skim milk solution at room temperature for 60 min to eliminate most of the nonspecific hybridizations. The membranes were subsequently immersed in 5% bovine serum albumin (BSA) in the presence of the polyclonal rabbit anti-cleaved caspase-3 antibody (1:1000, Cell Signaling Technology, MA, United States), the monoclonal rabbit anti-Mettl14 antibody (1:1000, Cell Signaling Technology, MA, United States), the polyclonal rabbit anti-Mettl3 antibody (1:1000, Proteintech Group, Inc, IL, United States), the monoclonal mouse anti-Wtap antibody (1:1000, Proteintech Group, Inc, IL, United States) or the monoclonal mouse anti-Fto antibody (1:1000, Abcam, United Kingdom), the monoclonal rabbit anti-Alkbh5 antibody (1:1000, Abcam, United Kingdom), and the monoclonal mouse anti-GAPDH antibody (1:10000, CoWin Biosciences, China) overnight at 4°C and hybridized with a horseradish peroxidase (HRP)–labeled goat anti-rabbit/-mouse antibody (1:8000; TransGen Biotech, China) at room temperature for 1 h. The blots were washed with TBST and then visualized by enhanced chemiluminescence (ECL; Millipore, MA, United States) through a luminescent imaging workstation (Tanon, China). The amount of protein was proportional to the optical density (OD) intensity measured by the ImageJ software (National Institutes of Health, MD, United States).

### RNA Preparation and Quality Control

Total RNA was extracted from the kidney specimens using the TRIzol reagent (Invitrogen, MA, United States) following the manufacturer’s instructions. The quantity and quality of the total RNA were assessed by using a NanoDrop ND-1000 spectrophotometer (NanoDrop Technologies, DE, United States). The RNA integrity was determined by denatured agarose gel electrophoresis.

### m^6^A RNA Methylation Quantification

The total level of m^6^A in kidney tissue was determined by using the EpiQuik m^6^A RNA Methylation Quantification Kit (Epigentek, NY, United States) according to the manufacturer’s instructions. Briefly, 200 ng RNA isolated from kidney tissue was added to strip wells and incubated with the capture antibody and detection antibody in sequence. After treatment with enhancer solutions, the m^6^A content was quantified colorimetrically using a microplate spectrophotometer at 450 nm and calculated based on the formula provided by the manufacturer.

### Quantitative Real-Time PCR

The differential expression levels of the m^6^A-related genes *Mettl3, Mettl14, Fto, Wtap,* and *Alkbh5* were evaluated by quantitative real-time PCR (qPCR) in control and Cis-AKI mice. In brief, total RNA derived from kidney tissues was reverse transcribed into cDNA with SuperScript™ III Reverse Transcriptase (Invitrogen, MA, United States) according to the manufacturer’s protocol. The amplification reactions were then carried out on a QuantStudio™ 5 System (Applied Biosystems, MA, United States) with a 2X PCR master mix (Arraystar, MD, United States), which contained a 2× Master Mix (Arraystar, MD, United States), 10 μmol/L forward primer, 10 μmol/L reverse primer, and 2 μl cDNA. The reaction condition was as follows: 95°C for 10 min, followed by 40 cycles of 95°C for 10 s and 60°C for 60 s. The Ct values obtained against known concentrations of serially diluted PCR products of mRNAs were used to construct a standard curve. The concentration of the targeted mRNAs in each sample was calculated according to the standard curve. The mRNA expression levels were normalized using GAPDH as the internal reference, and the fold change between the two groups was then calculated. To confirm the validity of the microarray data, qPCR combined with methylated RNA immunoprecipitation was conducted to determine the expression level of ten selected RNAs, including mRNAs (Fosl1, Krt20, Cdkn1a, Psme2, Slc35f3, and Cndp1) and lncRNAs (RP24-267E6.2, RP23-478H15.3, RP23-149A5.3, and RP23-257O9.1). The immunoprecipitated RNA and input RNA were both converted to cDNA and subjected to an amplification process as described previously (Wang Y. et al., 2019). The sequences of the primers used in the qPCR experiments are listed in Table 1.

**TABLE 1:** Sequences of primers used for qPCR and MeRIP-qPCR analysis.

Name	Sequence	Product size (bp)
GAPDH	F:5′ CAC​TGA​GCA​AGA​GAG​GCC​CTA​T 3′	144
	R:5′ GCA​GCG​AAC​TTT​ATT​GAT​GGT​ATT 3′	
Mettl3	F:5′ GGT​TCG​TTC​CAC​CAG​TCA​TA 3′	155
	R:5′ CTA​GTA​GGT​GTA​TCC​CAT​CCA​G 3′	
Mettl14	F:5′ TGC​GAA​AGT​GGG​GTT​ACA​GAA 3′	172
	R:5′ ATG​AAG​TCC​CCG​TCT​GTG​CT 3′	
Fto	F:5′ CTT​CAC​CAG​GGA​GAC​TGC​TAT 3′	96
	R:5′ AGT​GGA​ACT​AAA​CCG​AGG​CT 3′	
Wtap	F:5′ AAA​GGT​CCG​ACT​GAG​TGA​AAC 3′	243
	R:5′ CAC​TCT​TGC​ATC​TCC​TGC​TCT 3′	
Alkbh5	F:5′ CGG​TCA​TCA​TTC​TCA​GGA​AGA​CA 3′	259
	R:5′ GAG​CTA​AAA​CTC​CCC​CTC​CG 3′	
Fosl1	F:5′ TGCCAAGCATCGACAGCA 3′	302
	R:5′ CAA​TTT​GTC​GGT​CTC​CGC​C 3′	
Krt20	F:5′ GCA​ATC​CAA​TTC​CAG​ACT​TGA 3′	172
	R:5′ CTT​AGC​ATT​GTC​AAT​TCG​CAG​G 3′	
Cdkn1a	F:5′ TAC​CGT​GGG​TGT​CAA​AGC​A 3′	171
	R:5′ GTG​CTG​TCC​CTT​CTC​GTG​A 3′	
Cxcl10	F:5′ TCT​CTC​CAT​CAC​TCC​CCT​TTA 3′	151
	R:5′ GCT​TCG​GCA​GTT​ACT​TTT​GTC 3′	
Cxcl1	F:5′ AAA​AGA​AGT​GCA​GAG​AGA​TAG​AGT 3′	234
	R:5′ GAG​ACC​AGG​AGA​AAC​AGG​GTT 3′	
Lrmda	F:5′ GCG​ACT​TGT​ACA​GAT​AAG​GAC​C 3′	156
	R:5′ TAA​GAC​GCA​GCC​AGT​GGA​G 3′	
Psme2	F:5′ TAC​TAC​TCA​CGG​TGG​ATG​TCT​T 3′	170
	R:5′ TGG​GAT​AGG​GAT​GTC​CAG​AG 3′	
Slc22a27	F:5′ AGC​TGT​AGG​AGT​CAT​TGG​GTT 3′	270
	R:5′ CCA​GGA​GTT​CCT​TAA​AAC​CTC 3′	
Slc35f3	F:5′ AGCGCCTGCAACAGTCCG 3′	178
	R:5′ CCA​CAC​CCC​AGA​AGA​CCT​TCT 3′	
Cndp1	F:5′ CCA​GGC​TAT​CAC​ACA​AAA​GAG​T 3′	194
	R:5′ TGC​CCG​TGG​ATC​AGT​AGA​CA 3′	
RP24-267E6.2	F:5′ CGT​CTG​TGG​TTC​AAA​CCA​ATC​A 3′	74
	R:5′ GGA​GCA​TCG​GGA​GTG​TAA​AG 3′	
RP23-478H15.3	F:5′ TCA​CTC​ATC​GCT​GTA​TTC​CC 3′	144
	R:5′ CAC​ACC​CTT​TAA​TAG​TGC​CAG​T 3′	
RP23-149A5.3	F:5′ CAC​CTG​CCT​GCT​ACC​ATA​CTT​C 3′	69
	R:5′ GAC​CCA​GTT​CCA​CAG​GGA​TAG 3′	
RP23-257O9.1	F:5′ ATA​GTT​AGG​GCA​CTA​ACT​TGC​C 3′	67
	R:5′ TGT​TCT​TGA​CCA​CTG​AGC​GT 3′	

### m^6^A Immunoprecipitation

Total RNA (3 μg) mixed with the m^6^A spike-in control reagent (Arraystar, MD, United States) was incubated with anti-m^6^A rabbit polyclonal antibody (Synaptic Systems, Germany) in an immunoprecipitation (IP) reaction system (50 mM Tris-HCl, 7.4 pH , 150 mM NaCl, 0.1% NP40, and 40 U/μl RNase Inhibitor (Enzymatics, MA, United States)) at 4°C for 2 h. Dynabeads™ M-280 Sheep Anti-Rabbit IgG (Invitrogen, MA, United States) was blocked with 0.5% bovine serum albumin (BSA) and then tumbled with the total RNA-antibody mixture prepared above. After binding to the total RNA, the beads were washed with IP buffer and Wash buffer (50 mM Tris-HCl, 7.4 pH , 50 mM NaCl, 0.1% NP40, and 40 U/μl RNase Inhibitor) several times. Finally, the enriched RNA was separated from the beads in Elution buffer (10 mM Tris-HCl, pH7.4, 1 mM EDTA, 0.05% SDS, and 40 U Proteinase K) and purified *via* acid phenol–chloroform extraction and the ethanol precipitation method.

### m^6^A RNA Microarray

The immunoprecipitated RNAs and RNAs in the supernatant were amplified separately as complementary RNAs (cRNAs) and labeled with Cy5 and Cy3 fluorescent dye using an Arraystar Super RNA Labeling Kit (Arraystar, MD, United States). The Cy3 and Cy5 marked cRNAs were mixed and purified by using an RNeasy Mini Kit (Qiagen GmbH, Germany). Following fragmentation by adding the blocking agent and fragmentation buffer, the cRNA mixture was combined with the hybridization buffer and then loaded onto the m^6^A-mRNA and lncRNA Epitranscriptomic Microarray slide (KangChen Bio-tech, China) for hybridization in an Agilent Hybridization Oven at 65°C for 17 h. The hybridized microarray slides were washed and scanned with the Agilent Scanner G2505C (Agilent Technologies, CA, United States).

### Microarray Data Analysis

Raw data were obtained with Agilent Feature Extraction software (version 11.0.1.1) by analyzing the scanned array images. After normalization of raw intensities of immunoprecipitated RNAs (Cy5-labeled) and RNAs in the supernatant (Cy3-labeled) with an average intensity of spike-in RNA, the probe signals flagged as present (P) or marginal (M) in at least four out of eight samples were retained for further analysis. The m^6^A-methylated RNAs having |fold changes| (|FC|) ≥2 and *p* values ≤ 0.05 between the control and Cis-AKI groups were identified as of the significantly differential expression. The false discovery rate (FDR) approach was used to correct the *p* value. Gene ontology (GO) is a bioinformatic method for annotating genes, gene products, and sequences to the underlying biological activities (Gene Ontology Consortium, 2006). The ontology can be divided into three nonoverlapping domains: the biological process (BP), cellular component (CC), and molecular function (MF). The potential functions of the differentially expressed m^6^A-methylated RNAs were predicted according to the functional annotation of the host genes. Enrichment analysis for GO terms was performed using the topGO package in R software for statistical computing. The pathway analysis based on Kyoto Encyclopedia of Genes and Genomes (KEGG) was utilized to identify the pathways significantly enriched in the parental genes of the differentially expressed m^6^A-methylated RNAs. The enrichment *p* value of the pathway analysis was calculated by Fisher’s exact test.

### Statistical Analysis

All data are expressed as the means ± SEM of three or more independent experiments. Student’s *t*-tests were used to determine the differences between the two groups. A *p* value ≤ 0.05 was defined as statistically significant. SPSS 20.0 software (SPSS Inc., IL, United States) was used to analyze the data.

## Result

### Cisplatin-Induced AKI and Renal Tubular Epithelial Cell Apoptosis

**FIGURE 1:**
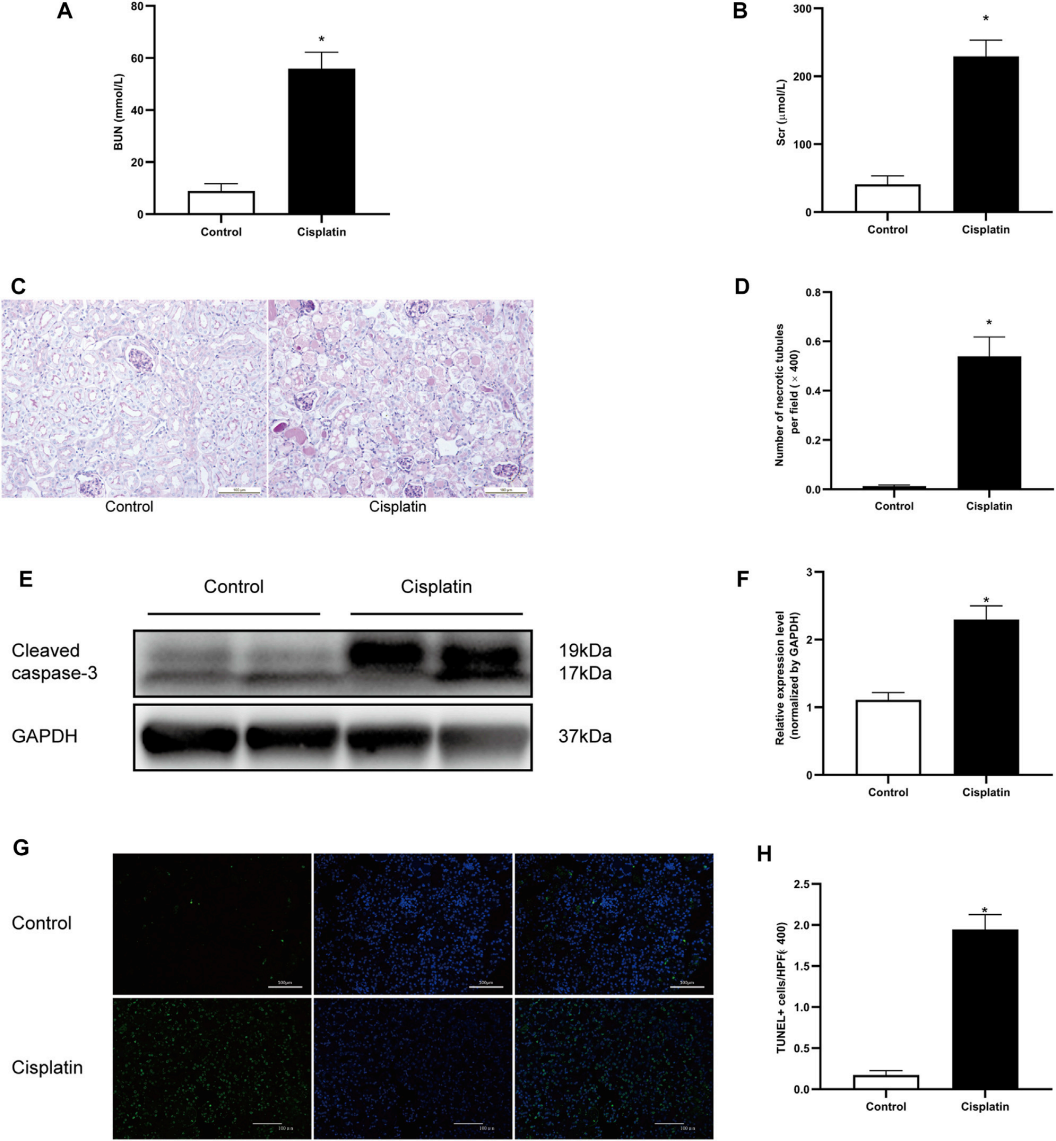
Cisplatin-induced AKI in mice. 129 male mice were treated with vehicle or cisplatin (20 mg/kg, intraperitoneal) and sacrificed on day 3 after cisplatin injection. **(A)** The histogram showing the level of blood urea nitrogen (BUN) in each group. *p < 0.05 vs. control group, *n* = 4 per group. **(B)** The histogram showing the level of creatinine in each group. *p < 0.05 vs. control group, *n* = 4 per group. **(C)** Periodic acid–Schiff (PAS) staining showing the histopathologic changes in the kidneys. Original magnification ×200; bar = 100 μm. **(D)** The histogram showing the number of necrotic tubules in the kidneys. Data are the mean ± SD of 20 random fields obtained in each kidney slice. *p < 0.05 vs. control group, *n* = 4 per group. **(E)** Representative immunostaining images showing cleaved caspase-3 expression in kidney tissues. GAPDH was used as a loading control for normalizing changes in specific gene expressions. **(F)** The histogram showing the level of cleaved caspase-3 protein expression that was calculated by densitometric analysis. *p < 0.05 vs. control group, *n* = 4 per group. **(G)** Representative micrographs showing the terminal deoxynucleotidyl transferase dUTP nick end labeling (TUNEL) staining among different groups. Original magnification ×200; bar = 100 μm; green: TUNEL; blue: DAPI. **(H)** Quantitative analysis of TUNEL staining positive cells among different groups. Data are the mean ± SEM of 20 random fields obtained in each kidney slice. *p < 0.05 vs. control group, *n* = 4 per group.

The blood urea nitrogen (BUN) and serum creatinine (Scr) were measured to evaluate the renal function of the mice. After 72 h of treatment with cisplatin, a significant increase of BUN and Scr was observed (Figures 1A,B). Pathologic analysis also showed more severe tubular injury after cisplatin exposure, as demonstrated by the acute and progressive tubular dilation, proteinaceous cast formation, and necrosis (Figures 1C,D). Consistent with these results, the expression level of cleaved caspase-3 and the number of apoptotic tubular cells were markedly increased in the cisplatin-treated mice compared with the controls (Figures 1E–H). Overall, these results demonstrated that a mouse model of AKI was established successfully.

### Cisplatin Increased the Total m^6^A Level and Modulated the m^6^A-Related Genes in the Mouse Kidneys

**FIGURE 2:**
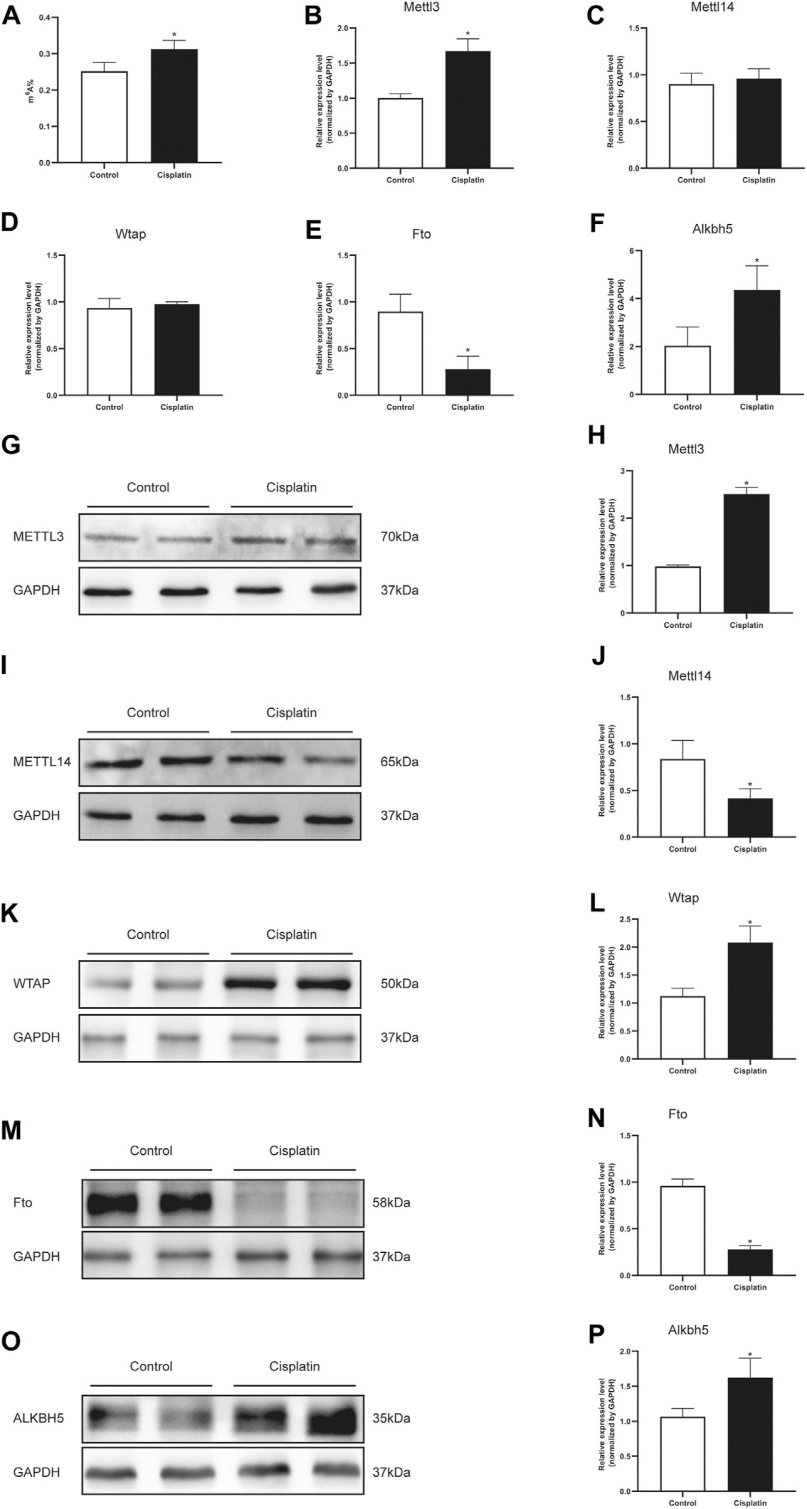
Cisplatin increased the total m^6^A level and modulated the m^6^A-related genes in the mouse kidneys. **(A)** Quantification of m^6^A abundance in total RNA isolated from the kidneys of male mice treated with vehicle or cisplatin. The histogram showing the levels of Mettl3 **(B)**, Mettl14 **(C)**, Wtap **(D)**, Fto **(E)**, and Alkbh5. **(F)** mRNA expression was quantified by qPCR analysis. GAPDH was used as a housekeeping gene for normalizing changes in specific gene expressions. *p < 0.05 vs. control group, *n* = 4 per group. Representative immunostaining images and densitometric analysis were performed to assess the protein expression of m^6^A-related enzymes: Mettl3 **(G and H)**, Mettl14 **(I and J)**, Wtap **(K and L)**, Fto **(M and N)**, and Alkbh5 **(O and P)** in kidneys of Cis-AKI groups compared with control groups. GAPDH was used as an internal control. Data were expressed as mean ± SEM and analyzed using Student’s *t*-test to determine the differences between the two groups. *p < 0.05 vs. control group, *n* = 4 per group.

The m^6^A RNA methylation level in mice treated with cisplatin was quantified using a colorimetric method. The results indicated that the RNA m^6^A content in kidney tissues treated with cisplatin was higher than those derived from control groups (Figure 2A). The mRNA expression levels of five major enzymes involved in m^6^A methylation including Mettl3, Mettl14, Wtap, Fto, and Alkbh5 were assessed by qPCR. Compared with that in the control group, the expression of Mettl3 and Alkbh5 was increased in the mice with cisplatin-induced AKI, whereas the expression of Fto was decreased (Figures 2B,E,F). The mRNA levels of Mettl14 and Wtap were similar in the control and AKI groups (Figures 2C,D). In addition, the expressions of the abovementioned five genes were further tested by western blot. The results demonstrated that the protein expressions of Mellt3, Wtap, and Alkbh5 were increased in response to cisplatin treatment, whereas the expressions of Mettl14 and Fto were decreased (Figures 2G–P).

### Cisplatin Altered m^6^A Modification Profiles in the Kidney

**FIGURE 3:**
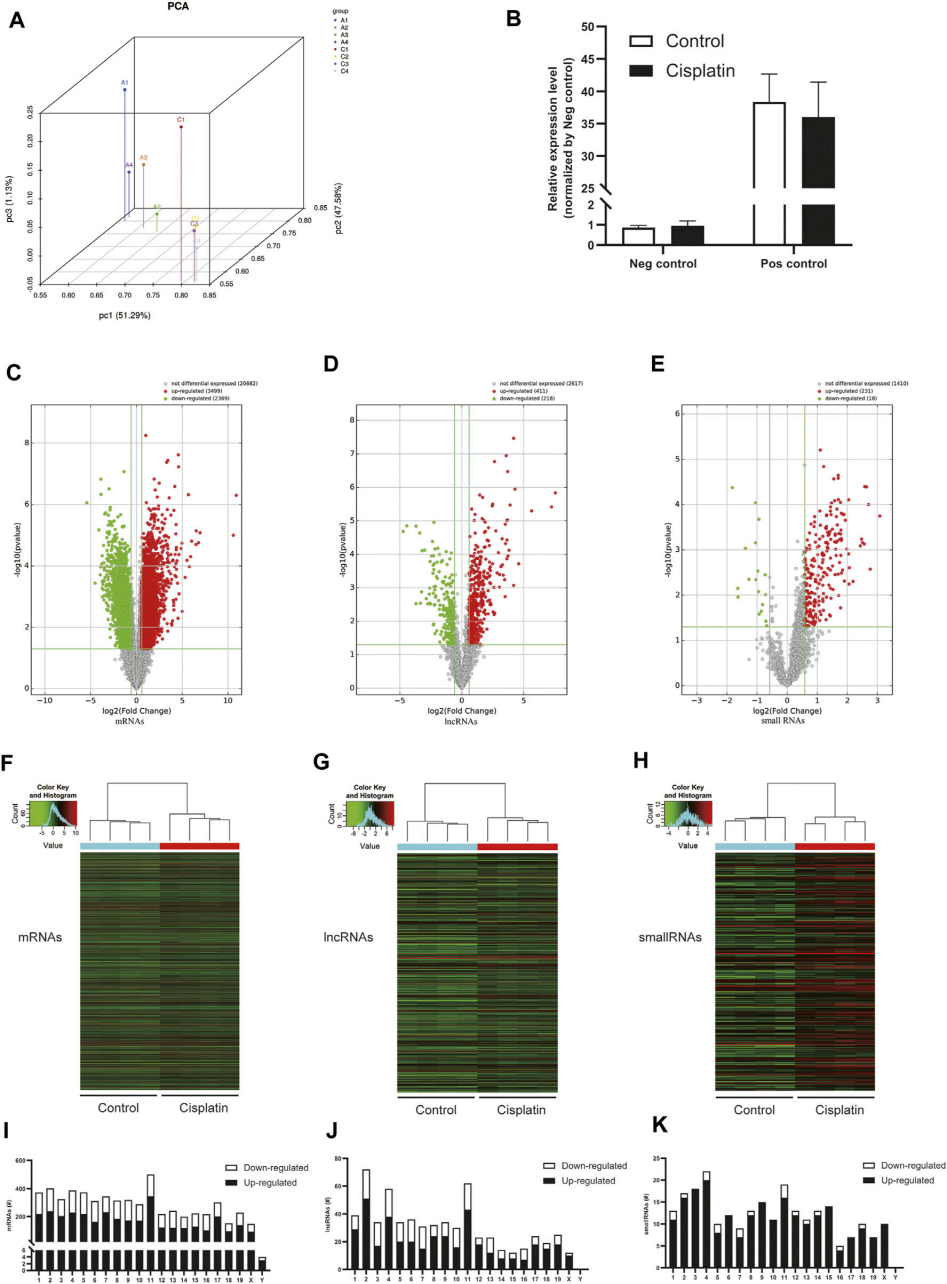
Cisplatin altered m^6^A modification profiles in the kidney. **(A)** Principal component analysis (PCA) was performed to categorize the differentially m^6^A modified transcripts between Cis-AKI and control groups (A: Cis-AKI; C: control; *n* = 4 per group). **(B)** The histogram showing the expression level of positive (Pos) and negative (Neg) spike-in RNA controls that were quantified by qPCR analysis (*n* = 4 per group); the volcano plots of mRNAs **(C)**, lncRNAs **(D)**, and small RNAs **(E)** expressions between Cis-AKI groups and control groups are shown. The spots represent upregulated (red) and downregulated (green) expressions of RNAs between the two groups. The heatmaps of the differentially m^6^A modified mRNAs **(F)**, lncRNAs **(G)**, and small RNAs **(H)** in Cis-AKI groups and control groups are shown. The distributions of altered mRNAs **(I)**, lncRNAs **(J)**, and small RNAs **(K)** among mouse chromosomes are shown.

To clarify the transcript-specific m^6^A modifications induced by cisplatin, we characterized the immunoprecipitated m^6^A methylated RNAs derived from the kidneys of Cis-AKI mice and their littermate controls. Principal component analysis (PCA) is a ubiquitous technique for data analysis and dimensionality reduction. The three-dimensional PCA clustering results calculated from the methylation data revealed distinct clustering and segregation between the individual samples of control and AKI groups (Figure 3A). The percentage of variance associated with PC1, PC2, and PC3 is 51.29, 47.58, and 1.13%, respectively (Figure 3A). These results indicated that the microarray profiles of the methylated RNA samples were rigor and reproducible. An RNA spike-in, designed to hybridize with a specific control probe on the target array, was used to measure sensitivity, accuracy, and biases in our microarray experiments for internal technology assessments (Jiang et al., 2011). As shown in Figure 3B, we observed that there was a similar change in the m^6^A enrichment of positive and negative spike-in controls between control and AKI groups, hence implying a rigorous analysis process. A total of 31,455 transcripts were identified in the four pairs of samples, including 26,550 mRNAs, 3,246 lncRNAs, and 1,659 small RNAs (pri-mRNA, pre-mRNA, snoRNA, and snRNA). The alteration in methylated RNAs with statistical significance between health and AKI mice was demonstrated by a volcano plot (Figures 3C–E). Based on the fold change of differentially methylated RNAs, hierarchical clustering was employed to show the distinguishable expression pattern of methylated transcripts between the two groups (Figures 3F–H). In response to cisplatin, a total of 618 mRNAs were observed to be differentially methylated by fold change ≥2.0 and *p* < 0.05, including 322 upregulated and 296 downregulated. In addition, 98 lncRNAs were detected to be differentially methylated by fold change ≥2.0 and *p* < 0.05 in cisplatin-treated mice compared with saline control animals, with 60 upregulated and 38 downregulated. It is noteworthy that the magnitude of change in methylation in mRNAs was larger than that in lncRNAs. Furthermore, 14 small RNAs were found to be differentially hypermethylated by fold change ≥2.0 and *p* < 0.05 in cisplatin-treated mice compared with control mice, with 14 upregulated and none downregulated. The top 20 altered methylated mRNAs, lncRNAs, and small RNAs are listed in Tables 2–4. The distributions of altered methylated transcripts in mice revealed that the differentially modified mRNAs were transcribed from all chromosomes, while the lncRNAs and small RNAs with differential methylation were transcribed from all chromosomes except chromosome Y (Figures 3I,J).

**TABLE 2:** Top 20 differently methylated mRNAs in the kidneys after cisplatin treatment.

Transcript_ID	Log_2_ (fold change)	Regulation	Chromosome	RNA length	Transcript_type	Gene symbol	*p* value	FDR
ENSMUST00000025850	10.90171777	Up	chr19	1,707	protein_coding	Fosl1	5.04959E-07	0.000893777
ENSMUST00000017743	10.58235631	Up	chr11	1,927	protein_coding	Krt20	1.01209E-05	0.003049634
ENSMUST00000023829	6.950454632	Up	chr17	1,910	protein_coding	Cdkn1a	8.37413E-06	0.002850425
ENSMUST00000044687	6.784580325	Up	chr12	1,093	protein_coding	Ifi27l2b	1.75887E-05	0.004967059
ENSMUST00000092426	6.533305034	Up	chr9	1,087	protein_coding	Ccdc153	7.2375E-06	0.003512516
ENSMUST00000127683	6.501754033	Up	chr2	445	protein_coding	RP23-430H1.2	2.02934E-05	0.002668828
ENSMUST00000100700	6.031748629	Up	chr5	1,233	protein_coding	Gm10382	1.56582E-05	0.003616034
ENSMUST00000038816	5.920444897	Up	chr5	1,094	protein_coding	Cxcl10	4.99512E-05	0.003415904
ENSMUST00000049209	5.776601971	Up	chr5	1,801	protein_coding	Gc	0.000105077	0.0062421
ENSMUST00000086370	5.681891497	Up	chr2	812	protein_coding	RP23-430H1.2	4.81714E-07	0.000893777
ENSMUST00000180430	−5.4136623	Down	chr5	13,125	protein_coding	Ksr2	8.83534E-07	0.001234623
ENSMUST00000029632	−4.51302473	Down	chr3	5,351	protein_coding	Lrat	0.000365844	0.010950184
ENSMUST00000159777	−4.09666987	Down	chr14	761	protein_coding	Lrmda	6.71672E-06	0.002511676
NM_001253358	−3.95059022	Down	chr14	5,063	protein_coding	Kcnma1	0.000196399	0.008396774
ENSMUST00000159687	−3.89483022	Down	chr14	564	protein_coding	Psme2	0.000109443	0.006316784
uc007suc.1	−3.88318978	Down	chr14	3,041	protein_coding	Erc2	1.5033E-07	0.000498909
ENSMUST00000162484	−3.87351184	Down	chr9	3,606	protein_coding	Cntn5	4.67443E-07	0.000893777
ENSMUST00000029833	−3.78628815	Down	chr3	2,652	protein_coding	Lrriq3	8.61405E-05	0.005739289
ENSMUST00000182102	−3.75097078	Down	chr19	1,801	protein_coding	Slc22a27	0.000532007	0.013078513
ENSMUST00000098441	−3.73934763	Down	chr9	8,781	protein_coding	Col6a6	4.69398E-05	0.004865821

**TABLE 3:** Top 20 differently methylated lncRNAs in the kidneys after cisplatin treatment.

Transcript_ID	Log_2_ (fold change)	Regulation	Chromosome	RNA length	Transcript_type	Gene symbol	*p* value	FDR
ENSMUST00000189460	7.550857116	Up	chr1	548	lncRNA	RP24-267E6.2	1.4627E-06	0.000787674
ENSMUST00000136927	7.244660674	Up	chr11	876	lncRNA	RP23-478H15.3	3.82383E-06	0.001034346
ENSMUST00000150329	5.639433808	Up	chr11	4,063	lncRNA	Egfros	5.06678E-06	0.00117477
ENSMUST00000132010	4.596570731	Up	chr4	5,432	lncRNA	Trp53inp1	0.000192292	0.007285261
ENSMUST00000192285	4.299689008	Up	chr1	2,763	lncRNA	RP23-349L18.2	1.1406E-06	0.000740479
ENSMUST00000131854	4.190638957	Up	chr1	517	lncRNA	Atf3	0.000850584	0.016146176
ENSMUST00000159701	4.187198822	Up	chr1	915	lncRNA	AC139673.3	3.41929E-08	0.00011099
ENSMUST00000216917	3.901038923	Up	chr9	3,485	lncRNA	RP23-110E20.5	3.38838E-06	0.001034346
ENSMUST00000189987	3.817308354	Up	chr10	1,095	lncRNA	Sobp	7.55471E-05	0.005004612
ENSMUST00000134701	3.730540579	Up	chr3	1,581	lncRNA	Dnajb4	0.0001113	0.005943006
ENSMUST00000189802	−4.73050701	Down	chr1	871	lncRNA	RP23-149A5.3	2.07759E-05	0.002325468
ENSMUST00000219854	−4.43488047	Down	chr10	1,718	lncRNA	RP23-417F2.1	1.41542E-05	0.001875077
ENSMUST00000218333	−3.69175411	Down	chr10	2,231	lncRNA	RP23-257O9.1	0.002985554	0.035662156
ENSMUST00000209842	−3.68939357	Down	chr7	688	lncRNA	RP23-114A6.4	1.44414E-05	0.001875077
ENSMUST00000181623	−3.39644228	Down	chr19	2,054	lncRNA	C330002G04Rik	2.31177E-05	0.00236634
ENSMUST00000195137	−3.33047484	Down	chr3	2,713	lncRNA	RP23-227A12.5	0.002907356	0.035367798
ENSMUST00000218833	−3.24928997	Down	chr10	628	lncRNA	RP24-377N13.1	0.001323028	0.022299215
ENSMUST00000138767	−3.20394732	Down	chr6	1,996	lncRNA	RP23-99G18.2	7.83749E-05	0.005088098
ENSMUST00000187223	−2.98711362	Down	chr6	3,096	lncRNA	Pik3c2g	7.49369E-05	0.005004612
ENSMUST00000175787	−2.864803	Down	chr4	1,693	lncRNA	RP23-365O6.3	0.002115895	0.029477237

**TABLE 4:** Top 20 differently methylated smallRNAs in the kidneys after cisplatin treatment.

Transcript_ID	Log_2_ (fold change)	Regulation	Chromosome	RNA length	Transcript_type	Gene symbol	*p* value	FDR
MI0021886-pri-5	3.082208595	Up	chr18	100	pri-miRNA	pri-5-mmu-mir-6358	0.000179897	0.010532486
ENSMUST00000183609	2.761690244	Up	chr11	215	snoRNA	Gm24949	0.002669786	0.036156425
ENSMUST00000083278	2.706661683	Up	chr6	159	snRNA	Gm26264	0.000100255	0.010395193
ENSMUST00000157083	2.640817688	Up	chrX	139	snRNA	Gm23000	4.06993E-05	0.007782031
ENSMUST00000083033	2.581393655	Up	chr1	167	snRNA	Gm24405	4.00991E-05	0.007782031
ENSMUST00000083111	2.565886571	Up	chr3	165	snRNA	Gm24046	0.000740094	0.017293193
ENSMUST00000083224	2.50485157	Up	chr13	227	snoRNA	Gm25483	0.000691744	0.016851184
MI0000562-pri-3	2.476254101	Up	chr13	100	pri-miRNA	pri-3-mmu-let-7f-1	0.000576182	0.016672703
MI0026036	2.433478337	Up	chr1	147	pre-miRNA	mmu-mir-3535	0.000815825	0.018540472
ENSMUST00000104215	2.191764197	Up	chr4	191	snoRNA	Gm25419	0.004983237	0.052157759
MI0022927-pri-3	−1.82022939	Down	chr8	100	pri-miRNA	pri-3-mmu-mir-7077	4.22172E-05	0.007782031
MI0000400-pri-3	−1.6397752	Down	chr12	100	pri-miRNA	pri-3-mmu-mir-300	0.007064005	0.065853371
MI0026022	−1.63115661	Down	chr16	129	pre-miRNA	mmu-mir-8095	0.01101019	0.085457802
MI0022781-pri-3	−1.3878493	Down	chr11	100	pri-miRNA	pri-3-mmu-mir-6934	0.00092536	0.01920793
MI0004686	−1.26916795	Down	chr5	109	pre-miRNA	mmu-mir-702	0.004460451	0.048464681
ENSMUST00000157160	−1.0470791	Down	chr2	124	snoRNA	Gm23172	9.25779E-05	0.010254389
MI0022901-pri-3	−1.04504735	Down	chr7	100	pri-miRNA	pri-3-mmu-mir-7052	0.000700863	0.016851184
MI0022869-pri-3	−1.03746499	Down	chr4	100	pri-miRNA	pri-3-mmu-mir-7020	0.004550455	0.048939999
ENSMUST00000122791	−0.97593288	Down	chr1	104	snRNA	Gm25294	0.002930654	0.038283107
ENSMUST00000175481	−0.94951247	Down	chr14	107	snRNA	Gm25464	0.008336336	0.073656993

### GO and Pathway Analyses of the Host Genes of Differentially m^6^A Modified RNA

**FIGURE 4:**
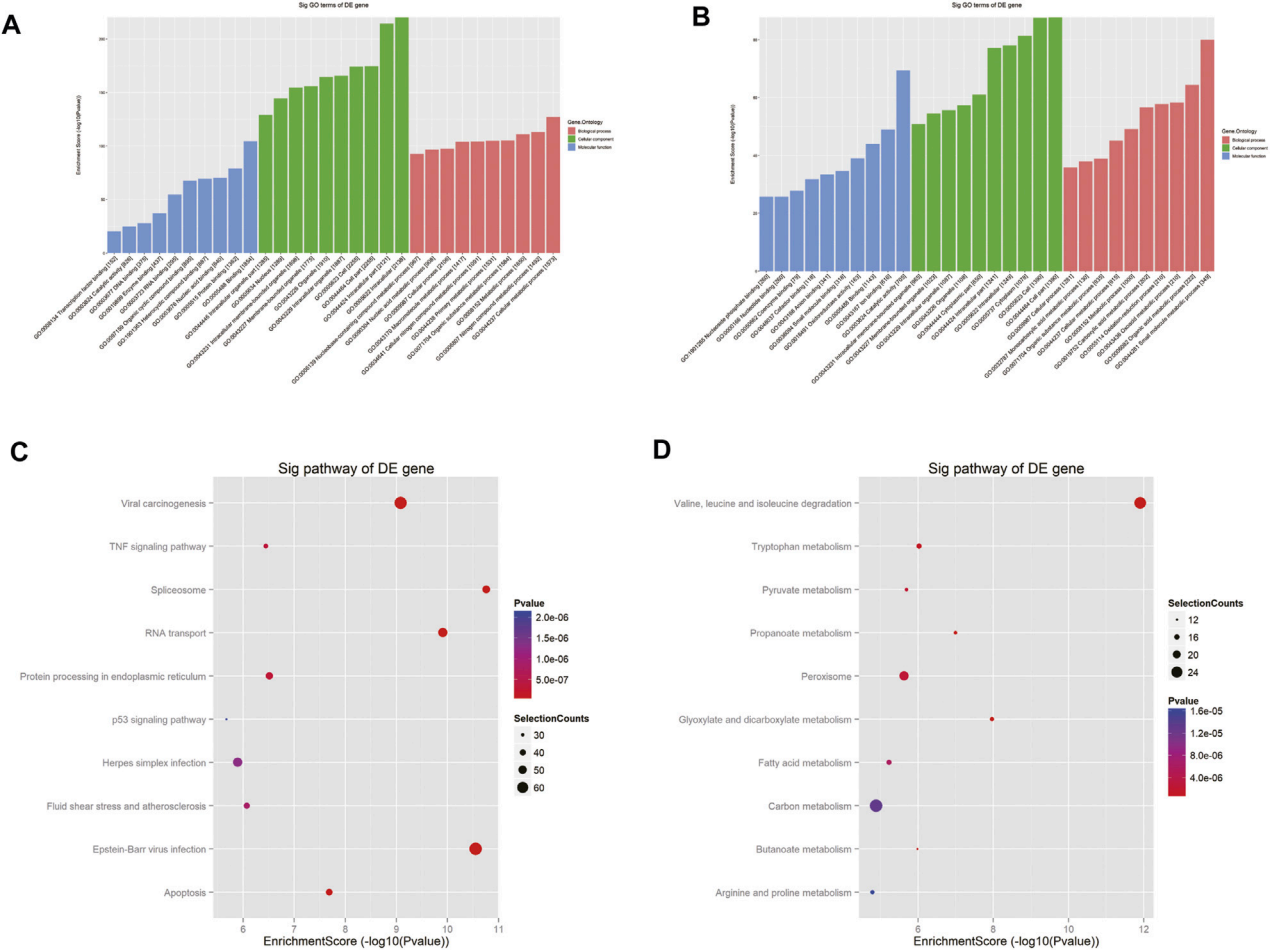
GO and pathway analyses of the host genes of differentially m^6^A modified RNAs. GO analysis on parental genes of the m^6^A hypermethylated **(A)** and hypomethylated **(B)** mRNA transcripts is shown. The vertical axis demonstrates the annotated functions of the target genes. The horizontal axis demonstrates the enrichment score (−log10 transformed *p* value) of each cluster. KEGG analysis on parental genes of the m^6^A hypermethylated **(C)** and hypomethylated **(D)** mRNA transcripts is shown. The horizontal axis shows the enrichment score (−log10 transformed *p* value). Node size: gene count; node color: *p* value.

To depict the physiological and pathological significance of m^6^A methylation in Cis-AKI, the host genes of the differentially methylated mRNA were investigated using GO and KEGG pathway analysis. GO analysis indicated that the upregulated methylated transcripts were enriched in the cellular metabolic process (ontology: biological process), intracellular process (ontology: cellular component), and binding (ontology: molecular function; Figure 4A). The downregulated methylated mRNAs were significantly associated with the small molecule metabolic process (ontology: biological process), cell part (ontology: cellular component), and catalytic activity (ontology: molecular function; Figure 4B). KEGG pathway analysis revealed 85 pathways related to hypermethylated genes, including spliceosome, Epstein–Barr virus infection, and RNA transport, and 78 pathways involved in hypomethylated genes, including valine, leucine, and isoleucine degradation, glyoxylate and dicarboxylate metabolism, and propanoate metabolism (Figures 4C,D).

### MeRIP-qPCR Verification of Differentially Methylated RNA Expression

**FIGURE 5:**
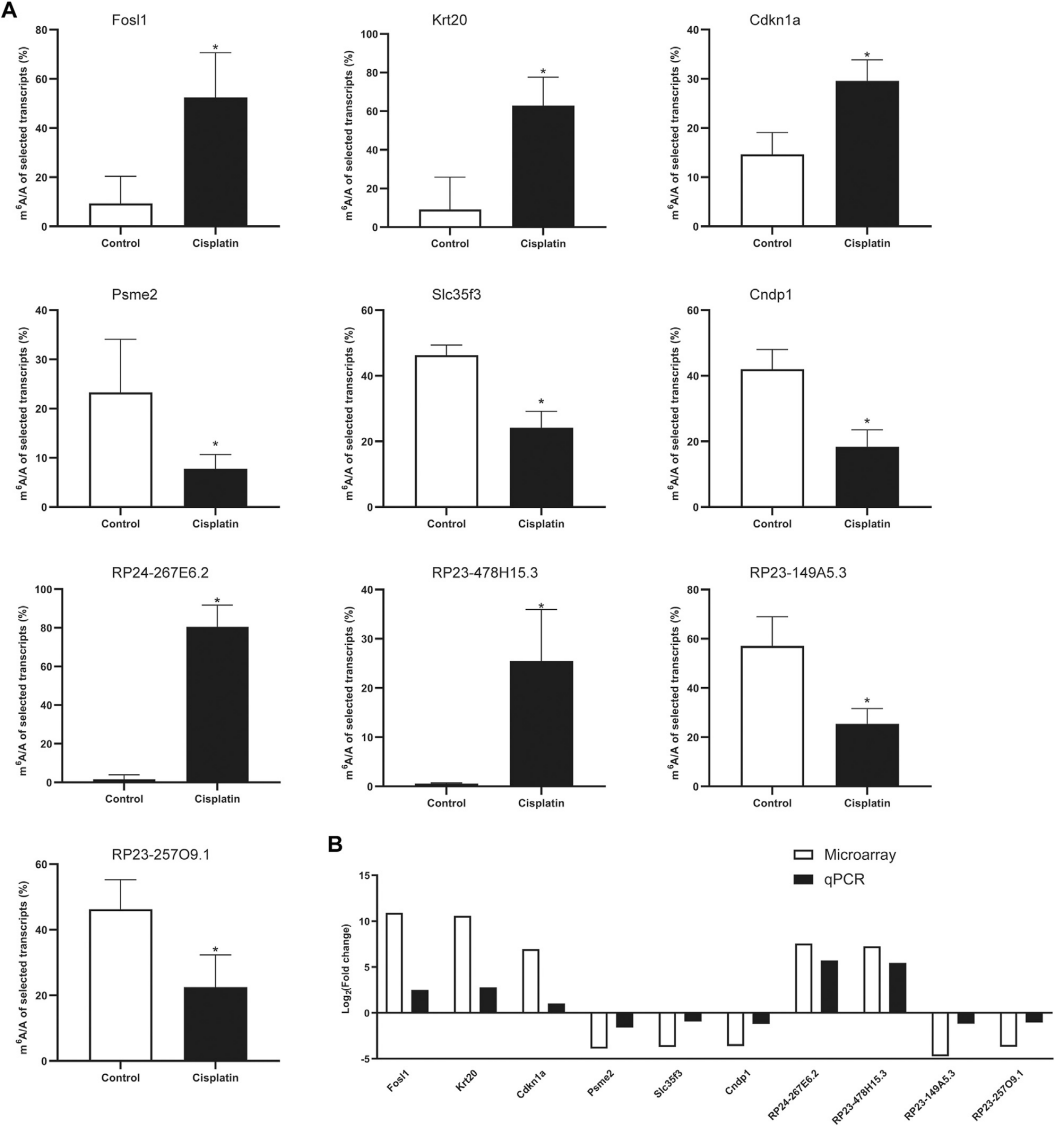
MeRIP-qPCR verification of differentially methylated RNA expression. **(A)** The m^6^A enrichment of selected mRNAs and lncRNAs was detected by the MeRIP-qPCR method. *p < 0.05, vs. control group, *n* = 4 per group. **(B)** The comparison between microarray data and MeRIP-qPCR results.

To validate the results of our microarray data, we performed m^6^A immunoprecipitation qPCR (MeRIP-qPCR) assays for six mRNAs and four lncRNAs in the Cis-AKI group and control group, respectively. The m^6^A methylation manifested a significant increase in Fosl1, Krt20, Cdkn1a, lncRNA RP24-267E6.2, and lncRNA RP23-478H15.3 in the kidney tissues exposed to cisplatin compared with those treated with saline (Figure 5A), whereas the m^6^A methylation levels of Psme2, Slc35f3, Cndp1, lncRNA RP23-149A5.3, and lncRNA RP23-257O9.1 were significantly decreased (Figure 5A). The abovementioned results exhibited a similar tendency of m^6^A methylation changes between microarray data and qPCR analysis (Figure 5B).

## Discussion

Nephrotoxicity is the most important and dose-limiting adverse event associated with the use of cisplatin that restricts its chemotherapeutic application. Although extensive research has suggested potential mechanisms that may be responsible for acute kidney injury induced by cisplatin, the molecular details are not yet fully understood (Agarwal et al., 2016; Ronco et al., 2019; Uber and Sutherland, 2019). m^6^A modifications in RNAs are recently recognized as functional regulators in a wide range of diseases, including neurological disorders, cancers, diabetes mellitus, leukemia, and coronary heart disease (Jonkhout et al., 2017; Vu et al., 2017; Kadumuri and Janga, 2018; Wang Y. et al., 2019; Mathiyalagan et al., 2019). However, much less is known about the role of m^6^A methylation in Cis-AKI. In the current study, we found that the m^6^A expression pattern is altered in the mouse kidneys after cisplatin treatment with the help of microarray technology. First, the m^6^A level in the kidney tissue was found to be higher in Cis-AKI mice than in littermate controls. Consistent with this result, the protein level of Mettl3 and Wtap was increased, and the protein level of Fto was decreased. Interestingly, the protein levels of Mettl14 and Alkbh5 exhibited an inverse trend compared with the change in total m^6^A levels. Furthermore, a total of 618 mRNAs and 98 lncRNAs were significantly differentially regulated and might be implicated in multiple biological processes and pathways. These findings provided an important opportunity to advance the understanding of the role of the m^6^A RNA modification in Cis-AKI.

An accumulating body of evidence suggests that the m^6^A modification is involved in the initiation and progression of kidney injury and repair. A recent study showed that the m^6^A level was elevated in renal tubular epithelial cells after TGF-β1 treatment, suggesting that m^6^A participated in the process of renal fibrosis (Liu P. et al., 2020). In addition, the m^6^A methylated RNA was also increased significantly in a mouse model of renal IRI (Xu Y. et al., 2020). Furthermore, the kidney biopsies from patients with AKI had remarkably higher m^6^A methylated RNA levels than those of patients with non-AKI (Xu Y. et al., 2020). Moreover, the m^6^A level was positively correlated with serum creatinine levels in these patients (Xu Y. et al., 2020). However, in an animal model of AKI induced by unilateral ureteral obstruction (UUO) operation, m^6^A methylation in adult mouse kidneys was found to decrease and showed a similar change of the expressions of Mettl3 and Mettl14 (Li X. et al., 2020). Taken together, these studies support the notion that m^6^A methylation had different regulatory functions in different types of AKI. In the current study, m^6^A methylated levels in total RNA were increased in the kidney tissues after cisplatin treatment. We further revealed that the expression patterns of m^6^A-modified RNAs changed significantly in response to cisplatin. Similar to our results, a recent study also found differences in the expression profile of m^6^A methylation in the kidney tissues after cisplatin treatment (Shen J. et al., 2020). The parent genes of the differentially expressed m^6^A methylated mRNAs, such as *Krt20, Ccdc85b*, and *Serpina3n* were found to be upregulated in both studies, while *Ctnna2, Afm, Pzp,* and *Dgkg* were downregulated. Due to the differences in the methods (microarray vs. RNA sequencing) and experimental animal species (129 vs. C57BL/6), the expression patterns of m^6^A-modified mRNAs in the two studies were not entirely consistent. However, the conclusions of the two studies are consistent, suggesting that m^6^A methylation may play a crucial role in the pathophysiologic process of Cis-AKI.

M^6^A is a dynamic and reversible modification that is modulated by the adenosine methyltransferases complex, including Mettl3, Mettl14, and Wtap, and m^6^A demethylases, such as Fto and Alkbh5 (Vu et al., 2019). In the present study, Mettl3 and Wtap were found to be upregulated in response to cisplatin. These alterations were correlated with the increased m^6^A modification. By contrast, the protein level of Mettl14 was decreased after cisplatin treatment, which was inconsistent with the trend of changes of the total m^6^A levels. Similarly, a recent study demonstrated that the abundance of m^6^A is partially correlated with the expression level of their writers and erasers (Liu J. et al., 2020). The authors found that the expressions of Mettl3 and Wtap, but not Mettl14, showed positive correlations with the m^6^A signal variation in human tissues (Liu J. et al., 2020). Thus, our unanticipated results suggested that the synthetic activity of the METLL3 and Wtap may exceed the effect of the downregulation of Mettl14, thus collectively contributing to the elevation of m^6^A modification in Cis-AKI. Nevertheless, the underlying mechanisms are still under scrutiny. The recent work by Panneerdoss S et al*.* revealed that Alkbh5/Mettl14 affected each other’s expression and restrained the translational stability of m^6^A reader YTHDF3, which, in turn, inhibited RNA demethylase activity and determined the m^6^A abundance of target genes (Panneerdoss et al., 2018). Therefore, this study outlines a critical role for Alkbh5 in the regulation of Mettl14 expression, which might be a potential mechanism for the inconsistency in the expression levels of Mettl3/Wtap and Mettl14, and ultimately leads to the total abundance of m^6^A upregulation.

Previous research has established that the adenosine methyltransferase complex is involved in a variety of biological processes, including cell cycle, cell proliferation, cell apoptosis, inflammation response, and metabolism (Liu S. et al., 2020). In mouse renal tubular epithelial cells, Mettl3 overexpression alleviated the colistin-induced renal injury by reducing apoptosis and enhancing the ability of antioxidative stress by the regulation of Nrf2, HO-1, and Keap1 (Wang J. et al., 2019). These results reflect those of Fanhang Meng et al*.* who also found that suppressing Mettl3 could lead to the decreased expression of cleaved caspase-3, resulting in the reduction of NRK-52E cell apoptosis (Meng et al., 2020). On the contrary, knocking down the expression of Mettl3 could inhibit the viability, proliferation, and migration potential of HK2 cells and, thus, attenuate the TGF-β1–induced epithelial–mesenchymal transition (EMT), suggesting that Mettl3 might play a detrimental role in the process of renal fibrosis (Liu P. et al., 2020). In addition to Mettl3, the expression of Mettl14 was found to consistently increase in the kidney biopsies from patients with AKI, compared with those from patients with non-AKI (Xu Y. et al., 2020). Moreover, loss of function of Mettl14 could mitigate the IRI-induced kidney injury through enhancing YAP1-TEAD signaling (Meng et al., 2020). Consistently, overexpression of Mettl14 was reported to significantly increase the expression of cleaved caspase-3 and promote apoptosis in HK2 cells with cisplatin treatment (Zhou P. et al., 2019). Therefore, the key members of the m^6^A writers, Mettl3 and Mettl14, might play a critical role in the development of AKI.

*Fto*, identified as the first obesity-susceptibility gene, has been repeatedly linked to obesity traits, insulin resistance, and type 2 diabetes (Dina et al., 2007; Frayling et al., 2007; Spoto et al., 2012; Loos and Yeo, 2014). Variants in this gene were subsequently reported to associate with the risk of chronic kidney disease (CKD), reduced estimated glomerular filtration rate (eGFR) in type 2 diabetes patients, and all-cause mortality in predialysis and dialysis patients (Franceschini et al., 2012; Hubacek et al., 2012; Spoto et al., 2012; Chen et al., 2013). This gene codes for an enzyme that selectively removes the methyl group from target RNAs, and by this mechanism, it may regulate epigenetic modifications of relevant genes to determine the cell fate. For example, a recent study showed that Fto-dependent m^6^A demethylation enhanced the expression of antiapoptotic genes and subsequently led to cell survival (Yan et al., 2018). Another study demonstrated that inhibition of Fto would increase m^6^A levels and aggravate the renal damages through p53-mediated apoptosis pathways (Zhou P. et al., 2019). On the contrary, several reports found that Fto was increased after ureteral obstruction and renal fibrosis (Wang C. Y. et al., 2016; Li X. et al., 2020). Furthermore, deficiency of the *Fto* gene attenuated the fibrogenic responses induced by ureteral obstruction in the kidney (Wang C. Y. et al., 2016). Moreover, recent evidence showed that Fto affected mitochondrial content and fat metabolism by modulating m^6^A levels in hepatocytes (Kang et al., 2018). Similarly, Changshui Zhuang and colleagues found that Fto influenced mitochondria integrity through regulating mitochondrial fusion, fission, and biogenesis (Zhuang et al., 2019). Furthermore, extensive research has revealed that improvement of mitochondrial function could alleviate cisplatin-induced nephrotoxicity (Li M. et al., 2020; Yu et al., 2020). In the present study, Fto was found to decrease in kidney tissue after cisplatin treatment and correlate with increased m^6^A modification. Therefore, Fto and the subsequent m^6^A modification might be involved in the pathological process of AKI.

Concerning another primary m^6^A demethylase, Alkbh5, there are plenty of studies that described its pivotal functions in multiple pathophysiological processes, such as proliferation, apoptosis, and autophagy (Wang et al., 2020). Recently, investigators have demonstrated that Alkbh5 was involved in neuronal apoptosis by affecting neurons’ methylation (Xu K. et al., 2020). Contrary to expectations, this study found that the increasing trend of RNA m^6^A modification was in parallel with the paradoxical upregulated expression of Alkbh5 in the cerebral cortex of rats after cerebral IRI (Xu K. et al., 2020). In accordance with their results, we found that the increased total abundance of m^6^A was consecutive to the increase of Alkbh5 expression after cisplatin treatment. It is difficult to explain this result, but the authors believed that it might be related to the compensatory effect of oxidative stress on Alkbh5 (Xu K. et al., 2020). Moreover, a recent study indicated that an increase in Alkbh5 might be compensated for the loss of Fto in the failing hearts (Mathiyalagan et al., 2019). Another possible explanation for these unexpected results may be the involvement of m^6^A writers and readers. As mentioned above, there was crosstalk between Mettl14 and Alkbh5, which is manifested as that knocking down of Mettl14 resulted in significantly reduced levels of Alkbh5, while silencing of Alkbh5 decreased Mettl14 levels (Panneerdoss et al., 2018). In addition, the activity of Alkbh5 can be further regulated by YTHDF3, an m^6^A reader, which restrained the activity of RNA demethylase (Panneerdoss et al., 2018). Therefore, the inconsistency between m^6^A level and Alkbh5/Mettl14 expression in the present study might be due to the compensatory effect of oxidative stress and Fto, as well as the crosstalk between Alkbh5 and Mettl14.

It is now well established from a variety of studies that cisplatin-induced nephrotoxicity is mediated through reactive nitrogen species (ROS), induction of proapoptotic and inflammatory pathways, and metabolic disorders (Perazella and Moeckel, 2010; Perazella, 2012; Perazella and Izzedine, 2015; Perazella, 2019). ROS plays a direct detrimental role in DNA and protein synthesis, cellular structure maintenance, and cell repair mechanisms (Perazella, 2019). The GO analysis results indicated that the altered m^6^A methylated transcripts were mainly associated with intracellular, binding, and cell part, which were closely related to the adverse effect of ROS, implying that the m^6^A modification might be essential for the oxidative stress response in AKI. In addition, further analysis demonstrated that the parent genes of the top three altered methylated mRNAs (*Fosl1*, *Krt20*, and *Cdkn1a*) participated in regulating apoptotic pathways (Torgovnick et al., 2018; Shen S. et al., 2020). A study conducted by Shen J et al. also found that genes with elevated methylation of m^6^A sites were significantly enriched in the cell death processes, such as apoptotic processes (Shen J. et al., 2020). Since apoptosis in renal tubular epithelial cells is a major feature of AKI, we would predict that m^6^A methylation has a pivotal role in tubular cell death.

Besides ROS and apoptosis, the metabolic disorder was identified as a new mechanism that leads to kidney function decline. The tubular epithelial cell, the most susceptible cell type to cisplatin in the kidney, combusts fatty acids to generate adenosine triphosphate (ATP) through oxidative phosphorylation for the high transport and reabsorption activities (Tammaro et al., 2020). Tran and colleagues recently showed that nicotinamide adenine dinucleotide (NAD^+^), an essential cofactor for fatty acid oxidation (FAO) (Szeto, 2017), was decreased in the renal tubular cells with IRI treatment, and this reduction in NAD^+^ may develop marked fat accumulation and renal function impairment (Tran et al., 2016). Our previous study also demonstrated that the levels of ATP, adenosine diphosphate (ADP), NAD^+^, nicotinamide adenine dinucleotide phosphate (NADP^+^), flavin mononucleotide (FMN), and other tricarboxylic acid cycle (TCA) products were significantly declined in response to cisplatin (Li M. et al., 2020). We further found that FAO improvement can attenuate Cis-AKI in mice (Li M. et al., 2020). In addition to fatty acid metabolism, Zhou et al*.* found that inhibitory *S*-nitrosylation of pyruvate kinase M2 (PKM2), a key enzyme of glycolysis, shunted metabolic intermediates and shifted metabolic flux from glycolytic to antioxidant pathways, leading to significant protection against declining kidney function in mice (Zhou H. L. et al., 2019). Furthermore, Legouis and colleagues showed that glucose production was impaired during AKI and this reduction in renal glucose due to the disorder of gluconeogenesis in proximal tubule cells was strongly associated with mortality in ICU patients with AKI (Legouis et al., 2020). This study highlights an unappreciated systemic role of renal glucose metabolism in kidney damage (Legouis et al., 2020). In cisplatin-induced AKI, a recent study indicated that genes with differently altered methylation of m^6^A sites were highly enriched in metabolic processes (Shen J. et al., 2020). Regarding amino acid metabolism, the levels of phenylalanine, allantoic acid, methylcysteine, gamma-aminobutyric acid, beta-alanine, and aminoadipic acid in the renal cortex of Sprague Dawley rats were increased significantly after intraperitoneal injection of endotoxin lipopolysaccharide (LPS) (Ping et al., 2019). Metabolic pathway analysis revealed that taurine and hypotaurine metabolism, pantothenic acid and CoA biosynthesis, and phenylalanine metabolism were involved in the development of AKI in sepsis (Ping et al., 2019). In the present study, we found that the host genes of the differentially methylated mRNA were enriched in the metabolic process and amino acid degradation. Therefore, we concluded that m^6^A methylation might be essential for the development and progression of AKI, regarding the abovediscussed functions of metabolic process and amino acid metabolism during kidney damage.

## Conclusion

In conclusion, the current work identified the differential m^6^A methylome in the kidney in response to cisplatin treatment, indicating a potential relationship between m^6^A modification and the regulation of the pathophysiologic process in AKI. Mettl3, Mettl14, Wtap, Fto, and Alkbh5 might play a vital role in the epigenetic modification of transcripts in cisplatin-treated kidneys. Overall, the empirical findings in this study might provide novel therapeutic targets for AKI treatment by fine-tuning the m^6^A modification.

## Data Availability

The original contributions presented in the study are publicly available. These data can be found here: https://www.ncbi.nlm.nih.gov/geo/query/acc.cgi?acc=GSE165100, accession number: GSE165100.
